# Impact of Oxidative Stress on Sperm Quality in Oligozoospermia and Normozoospermia Males Without Obvious Causes of Infertility

**DOI:** 10.3390/jcm13237158

**Published:** 2024-11-26

**Authors:** Linji Chen, Yusaku Mori, Shogo Nishii, Miwa Sakamoto, Makoto Ohara, Sho-Ichi Yamagishi, Akihiko Sekizawa

**Affiliations:** 1 Department of Obstetrics and Gynecology, Showa University Graduate School of Medicine, 1-5-8 Hatanodai, Shinagawa, Tokyo 142-8555, Japan; gm21-l034@med.showa-u.ac.jp (L.C.); shogo241@med.showa-u.ac.jp (S.N.);; 2 Department of Diabetes, Metabolism, and Endocrinology, Showa University Graduate School of Medicine, 1-5-8 Hatanodai, Shinagawa, Tokyo 142-8555, Japan

**Keywords:** advanced glycation end products, male infertility, oxidative stress, spermatozoa

## Abstract

**Background**: Male factors contribute to approximately 50% of infertile couples. However, obvious causes remain unknown in many cases. This observational study aimed to investigate the associations of clinical and lifestyle parameters with sperm parameters. **Methods**: This study enrolled 41 men in infertile couples without obvious causes for male infertility from July 2023 to April 2024. Semen samples were evaluated for sperm number, motility, DNA fragmentation, and oxidative stress (OS) marker oxidation–reduction potential (ORP). Blood samples were analyzed for biochemical parameters, including advanced glycation end products (AGEs), and systemic OS marker diacron-reactive oxygen metabolites (d-ROMs). Skin-accumulated AGE levels were identified with an autofluorescence method. Lifestyle factors were assessed with a lifestyle questionnaire. **Results**: Most of the participants were under 40 years old and non-obese with normal clinical parameters. Multiple regression analyses revealed that body mass index, serum d-ROMs, and semen ORP levels were independently associated with decreased sperm number. Additionally, serum zinc and semen ORP levels were associated with sperm motility. Furthermore, serum zinc and high-density lipoprotein cholesterol levels were associated with sperm progressive motility and DNA fragmentation, respectively. The rest of the clinical and lifestyle factors, including skin-accumulated and serum AGE levels, were not correlated with any sperm parameters. Furthermore, serum d-ROM and semen ORP levels were not correlated with each other or any of the clinical and lifestyle factors. **Conclusions**: Our present study indicates that both systemic and local OS may be independently involved in sperm abnormality in healthy men without obvious causes for male infertility.

## 1. Introduction

The infertility rate has been increasing in many countries, and approximately one in every six individuals at a reproductive age is estimated to experience infertility in their lifetime [1,2]. Reportedly, male infertility is implicated in approximately half of the cases of couples unable to conceive [1,2]. Various biological and environmental factors can cause male infertility, but no obvious causes of infertility are reported to be observed in 30–50% of male patients [1,2]. Obesity and undesirable lifestyles, such as smoking habits and alcohol overconsumption, are suspected to be risk factors for male infertility, but the underlying mechanism for this association remains unclear [3,4,5].

Accumulating evidence has indicated that obesity and undesirable lifestyle facilitate advanced glycation end products (AGEs) formation and oxidative stress (OS) generation, both of which play pathogenic roles in the development and progression of various types of chronic non-communicable diseases, including atherosclerotic cardiovascular disease, diabetes, and chronic kidney disease [6,7,8]. AGEs are molecules formed through macromolecule nonenzymatic glycation, such as proteins, lipids, and nucleic acids [6,7,8]. The formation and accumulation have progressed under hyperglycemic and/or chronic inflammatory conditions, and diet is a major environmental source of AGEs in the human body [6,7,8]. AGEs have been shown to induce OS generation and inflammation reactions in many cell and tissue types through their interaction with their cell-surface receptor, a receptor for AGEs (RAGE) [6,7,8]. We have recently revealed that AGE–RAGE axis activation contributes to a decreased total number, motility, and viability of sperm in a mouse model of diabetes with obesity partly by inducing testicular OS and inflammation [9]. Furthermore, many clinical studies have revealed that OS can impair the male reproduction system [10,11,12,13,14,15]. These results indicate that AGEs and OS may be one of the markers for male infertility without obvious causes, which is related to obesity and undesirable lifestyle habits. However, the association of AGEs and OS with sperm abnormality in apparently healthy men who lack obvious causes for male infertility remains unclear.

Therefore, we conducted an observational clinical study enrolling men in couples with infertility who lacked obvious causes for male infertility. In the present study, we utilized serum diacron-reactive oxygen metabolites (d-ROMs) and semen oxidation–reduction potential (ORP) levels as systemic and local OS markers, respectively, and evaluated their correlations with various sperm parameters, such as total number, total and progressive motility, and DNA fragmentation in sperm. Here, we further examined the association of skin accumulation and circulating AGE levels with the decreased total number and impaired function in sperm, independent of OS.

## 2. Materials and Methods

### 2.1. Ethics Statement

This observational study was conducted at Showa University Hospital from July 2023 to April 2024. The Ethics Committee of Showa University reviewed and approved the present study protocol (Approval No: 2023-047-B, approval date: 18 July 2023).

### 2.2. Study Participants

This study enrolled men in couples with infertility who visited the Reproduction section of the Obstetrics and Gynecology Department at Showa University Hospital (Tokyo, Japan). The exclusion criteria were (1) men who were <20 years old; (2) those with a current or past history of pyospermia/azoospermia, testicular injury or infection, varicocele, vasal reconstruction, or excessive exposure to radiation/chemicals; (3) those with active malignant or inflammatory disease; (4) those with chronic diseases such as chronic lung or liver failure; and (5) those who were deemed inappropriate for inclusion as evaluated by their respective physicians.

### 2.3. Study Design

All participants were evaluated with routine clinical and physical examination, lifestyle habits questionnaire [16], semen and blood sample collection in non-fasting conditions, and skin-accumulated AGE measurement. The questionnaire comprised 12 multiple-choice questions: (1) exercise frequency, (2) smoking habit duration, (3) alcohol consumption frequency, (4) sleep duration, (5) mental stress degree, (6) vegetable consumption, (7) breakfast frequency, (8) overeating habit, (9) greasy food consumption, (10) processed food consumption, (11) sugary food consumption, and (12) vegetable-first eating habit [16]. Each question was scored from 1 (the worst) to 5 (the best), and the sum of the scores was utilized as an individual value. All procedures were performed following the ethical standards of Showa University policy on human experimentation and the Helsinki Declaration of 1964 and its later version. Informed consent was obtained from all subjects involved in this study.

### 2.4. Laboratory Measurements

Systemic OS levels were evaluated by measuring serum hydroperoxide levels with the d-ROMs test (Wismerll Company Limited, Bunkyo, Tokyo, Japan), as previously described [17]. Assay calibration and sample measurement were performed according to the manufacturer’s instructions. In brief, 20 µL of serum was gently mixed with the pre-warmed assay reagents in the disposable test cuvette, and the cuvette was then loaded into the manufacturer’s colorimetric analyzer. Serum d-ROM levels were automatically measured in 5 min. The d-ROMs test results were expressed in Caratelli Units (U.CARR) [17]. Serum AGE levels were identified with an enzyme-linked immunosorbent assay [18]. Serum glucose, lipids, zinc (Zn), and free testosterone levels were measured with an enzyme electrode method, colorimetric assay, atomic absorption spectrophotometry, and radioimmunoassay, respectively. Skin-accumulated AGE levels were measured on the dorsal side of the forearms with a non-invasive autofluorescence method with AGE-Reader™ Mu (Diagnoptics Technologies B.V., Groningen, The Netherlands), as previously reported [16]. Reference values at non-fasting conditions were as follows: glucose of <200 mg/dL; total cholesterol (TC) of <220 mg/dL; high-density lipoprotein cholesterol (HDL-C) of >40 mg/dL; triglycerides (TG) of <150 mg/dL; Zn of 80–130 µg/dL; and free testosterone of >11.8 pg/mL.

### 2.5. Sperm Parameter Measurement

Semen samples were collected by masturbation after 2–7 days of sexual abstinence and analyzed following the World Health Organization (WHO) laboratory manual for the examination and processing of human semen, sixth edition [19]. The total number, total motility, and progressive motility in sperm were measured under a light microscope. A single-blinded investigator (L.C.) conducted all microscopic measurements to avoid technical bias. Normal values for sperm parameters were defined as follows [19]: semen volume of >1.4 mL; sperm concentration of >160 × 10^6^/mL; sperm number of >390 × 10^6^/ejaculate; abnormal total motility of <42%; and abnormal progressive motility of <30%.

### 2.6. Semen Oxidative Stress Measurement

Local OS levels were evaluated by measuring the ORP of semen samples with the MiOXSYS™ analyzer (Aytu BioScience, Englewood, CO, USA), which measures electron transfer from reductants (antioxidants) to oxidants, reflecting the overall balance between oxidants and antioxidants. Higher static ORP levels indicate oxidative stress due to an imbalance between oxidant and antioxidant activity. Assay calibration and sample measurement were performed according to the manufacturer’s instructions. In brief, 30 µL of liquefied flesh semen was loaded into the disposable test sensor, which was pre-inserted into the MiOXSYS analyzer, and ORP levels were automatically measured in 5 min. Semen ORP levels were then normalized by dividing the measured ORP values by the sperm concentration (10^6^/mL) [11,12]. Reference value for semen ORP was <1.34 mV/10^6^ sperm/mL [11].

### 2.7. Sperm DNA Fragmentation Measurement

Sperm DNA fragmentation was evaluated using the terminal deoxynucleotidyl transferase dUTP nick end labeling (TUNEL) method, as previously reported with some modifications [20]. Sperm smears were incubated with 10 mM of dithiothreitol for 1 min and further with 10 mM of lithium diiodo-salicylate and 1 mM of dithiothreitol for 2 h, then fixed in 4% paraformaldehyde for 1 h, permeabilized with 2% Triton X-100 and 0.1% bovine serum albumin for 15 min, and incubated with the primary antibody (Product ID: 11684795910; Sigma-Aldrich Japan, Meguro, Tokyo, Japan) at 4 °C overnight. Nuclei were stained with 4′,6-diamidino-2-phenylindole (DAPI; Product ID: D1306; Thermo Fisher Scientific, Waltham, MA, USA). Sperm DNA fragmentation was presented as a percentage of TUNEL-positive sperm to DAPI-positive sperm. Reference value for sperm DNA fragmentation was <19.25% [21].

### 2.8. Statistical Analyses

JMP Pro statistical software version 17.0.0 (SAS Institution Inc., Cary, NC, USA) was used for statistical analyses. The normality of data distribution was tested with the Shapiro–Wilk test (the results were shown in Supplemental Table S1). Data with normal and non-normal distributions were expressed as mean ± standard deviation (SD) and median with 25 and 75 percentiles, respectively. Categorical data were presented as percentages. Simple and multiple stepwise regression analyses were conducted with dependent variables, including the total number, total motility percentage, progressive motility percentage, and DNA fragmentation percentage in sperm. Independent variables were age, body weight, waist, body mass index (BMI), serum d-ROMs, skin-accumulated AGEs, serum glucose, serum TC, serum HDL-C, serum TG, serum Zn, serum free testosterone, serum AGEs, current smoking habit, current drinking habit, total lifestyle habits questionnaire score, abstinence period, and semen ORP. Two groups were compared with unpaired *t*-test, Wilcoxon signed-rank test, or Fisher’s exact test, as appropriate. Statistical significance was set at *p*-values of <0.05.

## 3. Results

### 3.1. Background Clinical and Lifestyle Characteristics

The analyses in the present study included 41 participants. Table 1 presents the ages and medical histories of the study participants and their partners. Table 2 shows the background clinical and lifestyle characteristics of the study participants. Most of the participants were <40 years old with a BMI of <25.0 kg/m^2^. The serum d-ROM levels were close to the upper limit of the normal range, which is <300 U.CARR [22]. The skin-accumulated AGE levels were within the normal range after adjusting for ages [16]. The serum glucose, TC, HDL-C, TG, Zn, and free testosterone levels were within the normal range in non-fasting conditions. Of the study participants, <25% had current smoking habits, whereas >75% had current drinking habits. The detailed results of the lifestyle habits questionnaire are presented in Supplemental Table S2 as the background lifestyle parameters of the study participants.

### 3.2. Background Sperm Parameters

Table 3 shows the sperm parameters of the study participants. According to the WHO laboratory manual for the examination and processing of human semen, sixth edition [19], the total number, total motility, and progressive motility in sperm were impaired in some of the participants, accounting for 39.0%, 14.6%, and 12.2% of the study participants with abnormal values, respectively. Additionally, the semen ORP levels and sperm DNA fragmentation percentages of all the participants were below the reported cut-off values for male infertility [11,22].

### 3.3. Simple and Multiple Regression Analyses of Clinical and Lifestyle Variables Related to Sperm Parameters

Table 4 presents the simple regression analyses of the clinical and lifestyle variables that were related to the sperm parameters. Of the significant variables, body weight (inversely), BMI (inversely), serum d-ROM levels (inversely), abstinence period, and semen ORP levels (inversely) were correlated with the total sperm number; serum Zn and semen ORP level (inversely) with the total motility in sperm; serum Zn level with the progressive motility in sperm; and body weight (inversely), BMI (inversely), waist (inversely), and serum HDL-C level with the fragmentation of sperm DNA.

Table 5 demonstrates the multiple stepwise regression analyses with the same variables, excluding body weight and waist because body weight, BMI, and waist were closely correlated with each other. Hence, BMI was selected for the analyses to avoid multicollinearity. The BMI, serum d-ROM levels, and semen ORP levels were inversely associated with the total number of sperm. The serum Zn level and semen ORP levels (inversely) were independent correlates of the total motility in sperm. The serum Zn and HDL-C levels were the sole independent correlates with the progressive motility in sperm and the DNA fragmentation in sperm, respectively. Supplemental Table S3 shows the additional multiple stepwise regression analyses, including lifestyle factors as mandatory variables. The results for sperm total number and motility were similar to the ones without the mandatory variables, whereas there was no significant clinical variable associated with either sperm progressive motility or DNA fragmentation.

### 3.4. Comparison of Clinical, Lifestyle, and Sperm Parameters in Two Subgroups with Higher vs. Lower Serum d-ROM Levels

A subgroup analysis, focusing on serum d-ROM levels, was conducted. The participants were evenly categorized into two subgroups based on their serum d-ROM levels. The median serum d-ROM level of all the participants was 299.0 U.CARR, and the participants above or equal to this value were assigned to the higher d-ROMs group. Table 6 presents the clinical, lifestyle, and sperm parameters of these subgroups. The serum glucose levels were greater in the higher serum d-ROMs group, with no significant differences in other the clinical and lifestyle parameters between the two groups. The lower serum d-ROMs group exhibited a greater semen volume and total sperm number compared with the higher serum d-ROMs group. No significant differences were found in the other sperm parameters between the subgroups.

### 3.5. Simple Regression Analyses of Clinical and Lifestyle Parameters Related to Serum d-ROM and Semen ORP Levels

Next, we examined the associations between the clinical, biochemical, and lifestyle parameters, and the serum d-ROM and semen ORP levels. In the simple regression analyses, none of the clinical, biochemical, or lifestyle parameters were significantly associated with serum d-ROM or semen ORP levels. Furthermore, serum d-ROM and semen ORP levels were not correlated with each other (*p* = 0.50). Moreover, in the multiple regression analyses for BMI and serum Zn level, which were found to be associated with the total sperm number and sperm motility, serum TG levels and age were the only correlate for the BMI and serum Zn level; serum d-ROM or semen ORP levels were not significantly associated with either BMI or serum Zn levels. The results were the same in the multiple regression analyses, including the lifestyle factors as mandatory variables.

### 3.6. Comparison of Clinical, Lifestyle, and Sperm Parameters in Two Subgroups of Normospermia and Oligospermia

Finally, a subgroup analysis focusing on sperm phenotype was conducted. The participants with a total sperm number of >390 × 10^6^/ejaculate were assigned to the normospermia group, and the rest were assigned to the oligospermia group [19]. Supplemental Table S4 presents the clinical, lifestyle, and sperm parameters of these subgroups. The serum d-ROM levels tended to be higher in the oligospermia group than in the normospermia group; however, no significant differences were found in the other clinical and lifestyle parameters between the two groups. The oligospermia group exhibited lower sperm-related parameters and higher semen ORP levels compared with the normospermia group. Supplemental Tables S5 and S6 show the raw statistical data and correlation matrices for the serum d-ROM and semen ORP levels and sperm phenotype. The semen ORP levels were significantly correlated with the sperm phenotype.

## 4. Discussion

This study investigated the associations of clinical and lifestyle factors with sperm parameters in men in couples with infertility who lacked an obvious cause for male infertility. Most of the study participants were under 40 years old and non-obese with normal biochemical parameters. We revealed that the serum and semen OS levels assessed by d-ROMs and ORP, respectively, besides BMI, were independently associated with a decreased total number of sperm. Furthermore, semen OS levels were correlated with a reduced total motility in sperm. Serum Zn levels were positively associated with total and progressive motility in sperm among the other clinical and lifestyle parameters, whereas HDL-C levels were independently correlated with DNA fragmentation in sperm. The rest of the clinical and biochemical parameters and lifestyle habits, including skin-accumulated and serum AGE levels, were not correlated with any sperm parameters. Additionally, we revealed that serum d-ROM and semen ORP levels were not correlated with any of the clinical, biochemical, or lifestyle parameters. Furthermore, no correlation between serum d-ROM and semen ORP levels was found, which is consistent with previous studies [23,24]. Thus, our present study indicates that OS may be involved in sperm abnormality of health men without obvious causes for male infertility, and that semen OS may be independently associated with decreased number and motility in sperm, whereas systemic OS may be independently correlated with reduced sperm number.

Sperm are vulnerable to OS-induced damage because of their high polyunsaturated fatty acid content, antioxidant enzyme deficiency, and DNA repair capacity limitations [10,13]. Semen ORP has been generally used as a sperm OS indicator [11,12], and the levels have been higher in men with abnormal sperm quality than in those with normal sperm quality [11]. Furthermore, the same study [11] revealed that, in men with at least one abnormal sperm parameter, semen ORP levels were inversely correlated with semen parameters, such as sperm concentration, total count, total and progressive motility, and normal morphological form, and that the cut-off value for abnormal sperm quality was calculated as 1.34 mV/10^6^ sperm/mL. On the other hand, a recent study showed that semen ORP levels were not associated with sperm parameters in men with idiopathic male infertility [23]. However, the involvement of semen ORP in the decreased total number and impaired total and progressive motility in sperm remains unknown in participants with almost normal sperm parameters. In this study, >60% of participants had a normal number of sperm and motility and semen ORP levels that were below the reported cut-off value, but the semen OS levels were independently correlated with a decreased total number and motility in sperm. This result indicates that, even below the reported cut-off value, semen ORP levels are inversely associated with both sperm number and motility in healthy men.

Systemic OS has negatively affected fertilization ability in men, in addition to semen OS [14,15]. Plasma levels of malondialdehyde (MDA), a marker of lipid peroxidation [25], and leukocyte 8-hydroxy-2′-deoxyguanosine (8-OHdG), a marker of oxidative DNA damage were significantly higher in infertile men without obvious infertility causes compared with fertile men, both of which were correlated with each other [14]. Moreover, leukocyte 8-OHdG levels were inversely associated with sperm count and total and progressive motility in sperm, whereas plasma MDA levels were inversely associated with progressive motility in sperm [14]. Additionally, another study revealed that the plasma MDA and leukocyte OS levels were higher and the plasma antioxidant capacity was lower in patients with idiopathic male infertility than in age-matched normozoospermic controls, and the plasma MDA levels were inversely correlated with the total number and concentration of sperm in these patients [15]. The present study assessed systemic OS levels with the d-ROMs assay, which measures the total amount of hydroperoxides in serum [17,21]. We revealed in this study that serum d-ROM levels were correlated with a decreased total number of sperm, independent of semen ORP levels and other clinical parameters. In the subgroup analysis, the higher serum d-ROMs group demonstrated a lower semen volume and total sperm number and greater plasma glucose levels compared to the lower serum d-ROMs group, but there were no significant differences in the other clinical or biochemical parameters except for semen ORP levels between the two subgroups. The mean values in the higher vs. lower serum d-ROMs groups were 343 vs. 267 U.CARR, and serum d-ROM levels of <300, 300–320, and >320 U.CARR were considered to correspond to normal, borderline, and increased OS conditions, respectively [21]. Thus, our present results indicate that increased systemic OS may contribute to decreases in total sperm number, and that healthy men with serum d-ROM levels over 343 U.CARR may be considered as a high-risk group for a decreased sperm number. On the other hand, serum d-ROM levels were not associated with sperm motility or DNA fragmentation. Spermatogenesis is initiated from spermatogonia located in the basal membrane of seminiferous tubules, thereby being affected by systemic factors [26]. Conversely, spermatocytes produced from spermatogonia via mitosis are supported by the epithelium of Sertoli cells, which comprises the blood–testis barrier, and then differentiate into sperm, which cannot be affected by systemic factors [26]. Therefore, increased systemic OS assessed by higher d-ROM levels may affect sperm production rather than sperm functional maturation through a mechanism distinct from local OS.

Previous studies have revealed that obesity negatively affects male fertilization ability [5]. The present study revealed that the mean BMI of the study participants was 24.5 kg/m^2^, which falls within the non-obese range [27], but BMI was associated with a decreased total number of sperm, independent of the other clinical or biochemical factors. A higher BMI may become a marker of visceral obesity that could be related to adipokine disturbances and chronic inflammation [28], thereby contributing to a decreased total number of sperm in concert with systemic OS. Further, this study revealed that the mean serum Zn levels of the study participants were within the normal range, but Zn levels were positively correlated with total and progressive motility in sperm, which was consistent with a previous result [29]. Optimal BMI control and Zn supplementation may be potential therapeutic targets for ameliorating the fertilization ability in men with infertility without obvious causes. In this study, systemic or local OS levels were not significantly associated with BMI or serum Zn in the multiple regression analyses. The BMI and serum Zn levels of the study participants were almost within the normal range, which may partly explain the lack of significant associations among them.

The present study has several limitations. First, we could not find here a significant correlation between the sperm parameters and skin-accumulated AGE levels. The number of study participants in the present study was only 41, with normal levels of skin-accumulated AGE levels [16], which may partly explain the lack of their association. Additionally, other metabolic pathways or the dietary intake of AGEs may have confounded the present findings. Furthermore, semen contains a large amount of fructose that serves as a main energy source in sperm, the levels of which are approximately 300-fold higher than those in other body fluids [30]; thus, the AGE measurement tools utilized in this study may have not worked for detecting fructose-derived AGEs. Secondly, the exact reason for the association of HDL-C levels with DNA fragmentation, which was not consistent with the previous observations, is unknown [31,32]. We evaluated nutrient intake with the questionnaire, but not with food records; thus, dietary factors may have confounded the present results. Thirdly, serum d-ROMs and semen ORP were used as systemic and local OS markers, respectively, because (1) their assays are semi-automated, which contributes to the accuracy and reproducibility of the measurements, (2) their cut-off values for an increased OS condition and male infertility, respectively, are clearly shown in previous studies [11,12,21]. These points would be important for considering whether the present findings can be applicable to clinical settings of different facilities. However, it remains unclear whether these markers are the most accurate predictors of sperm abnormality in the present study population. Fourthly, in the present study, men under the age of 20 were left out, despite the fact that their inclusion could yield useful early-onset data. However, 19-year-old or younger men without obvious male infertility causes rarely visit hospitals with a complaint of infertility. Indeed, no one met this exclusion criteria during the enrollment period in this study. Finally, this is a cross-sectional study, and thus it cannot elucidate the causal relationship between systemic and semen OS and sperm abnormality.

## 5. Conclusions

Our present study indicates that both systemic and local OS may be independently involved in sperm abnormality in healthy men without obvious causes for male infertility. Since sperm number and motility are crucial factors for male fertilization ability, the simultaneous measurement of systemic and local OS may be useful to identify high-risk subjects for impaired fertilization ability in apparently healthy men without obvious causes for male infertility.

## Figures and Tables

**Table 1:** The ages and medical histories of the study participants and their partners.

Participants (Men)	Values	Partners (Women)	Values
Number	41	Number	41
Age (years old)	37.5 ± 6.3	Age (years old)	35.8 ± 4.6
Comorbid disease		Comorbid disease	
Hypertension, *n* (%)	2 (4.9)	Uterine fibroids, *n* (%)	10 (24.4)
Type 2 diabetes, *n* (%)	2 (4.9)	Ovulation dysfunction, *n* (%)	6 (14.6)
Hyperuricemia, *n* (%)	1 (2.4)	Endometriotic cyst, *n* (%)	3 (7.3)
Hypercholesterolemia, *n* (%)	1 (2.4)	Sexual dysfunction, *n* (%)	2 (4.9)
Depression, *n* (%)	1 (2.4)	Polycystic ovary syndrome, *n* (%)	1 (2.4)
		Uterus duplex, *n* (%)	1 (2.4)
		Graves’ disease, *n* (%)	1 (2.4)
		Hypothyroidism, *n* (%)	1 (2.4)
		Rheumatoid arthritis, *n* (%)	1 (2.4)

Data are expressed as mean ± standard deviation (SD).

**Table 2:** Background clinical and lifestyle characteristics of study participants.

Parameters	Values
Body weight (kg)	73.3 ± 11.5
BMI (kg/m^2^)	24.3 ± 3.3
Waist (cm)	87.8 ± 10.1
Serum d-ROMs (U.CARR)	306 ± 49.9
Skin AGEs (AF)	1.70 (1.60–1.83)
Serum glucose (mg/mL)	103 (99–119)
Serum TC (mg/dL)	193 (172–207)
Serum HDL-C (mg/dL)	49.5 ± 13.5
Serum TG (mg/dL)	108 (68–160)
Serum Zn (µg/dL)	81 (69–92)
Serum free testosterone (pg/mL)	12.6 ± 3.8
Serum AGEs (µg/dL)	0.12 (0.10–0.17)
Current smoking habit (%)	22.0
Current drinking habit (%)	75.6
Lifestyle habits questionnaire score	36.8 ± 7.4

Data are expressed as mean ± SD, median with 25 and 75 percentiles, and percentage for data with normal distribution, non-normal distribution, and category, respectively. BMI: body mass index, d-ROMs: diacron-reactive oxygen metabolites, AGEs: advanced glycation end products, TC: total cholesterol, HDL-C: high-density lipoprotein cholesterol, TG: triglycerides, Zn: Zinc.

**Table 3:** Background sperm parameters of study participants.

Parameters	Values
Abstinence period (days)	3.0 (2.8–7.0)
Semen volume (mL)	3.2 (2.4–3.8)
Sperm concentration (10^6^/mL)	136 (76–212)
Total sperm number (10^6^/ejaculate)	456 (253–682)
Total Sperm motility (%)	59.7 ± 17.5
Progressive motility in sperm (%)	51.6 ± 17.7
Semen ORP (mV/10^6^ sperm/mL)	0.22 (0.12–0.41)
Sperm DNA fragmentation (%)	4.5 (2.7–8.3)

Data are expressed as mean ± SD or median with 25 and 75 percentiles as appropriate. ORP: oxidation–reduction potential.

**Table 4:** Simple regression analyses of clinical and lifestyle parameters related to sperm parameters.

Sperm Parameters	Total Number		Total Motility		Progressive Motility		DNA Fragmentation	
Variables	*β*	*P*	*β*	*p*	*β*	*p*	*β*	*p*
Age (years old)		0.81		0.24		0.23		0.38
Body weight (kg)	−0.36 [−0.60–−0.06]	0.02		0.55		0.65	−0.37 [−0.61–−0.07]	0.02
BMI (kg/m^2^)	−0.39 [−0.63–−0.10]	0.01		0.35		0.40	−0.35 [−0.59–−0.05]	0.02
Waist (cm)	−0.27 [−0.54–−0.04]	0.08		0.50		0.56	−0.40 [−0.63–−0.11]	<0.01
Serum d-ROMs (U.CARR)	−0.46 [−0.67–−0.17]	<0.01		0.28		0.31		0.72
Skin AGEs (AF)		0.28		0.28		0.33		0.34
Serum glucose (mg/mL)		0.06		0.07		0.09		0.21
Serum TC (mg/dL)		0.32		0.74		0.64		0.73
Serum HDL-C (mg/dL)		0.85		0.54		0.69	0.40 [0.10–0.63]	0.01
Serum TG (mg/dL)		0.67		0.47		0.31		0.09
Serum Zn (µg/dL)		0.81	0.38 [0.08–0.62]	0.01	0.39 [0.09–0.62]	0.01		0.24
Serum free testosterone (pg/mL)		0.80		0.76		0.92		0.15
Serum AGEs (µg/dL)		0.36		0.22		0.30		0.33
Current smoking habit (%)		0.94		0.81		0.56		0.93
Current drinking habit (%)		0.73		0.98		0.83		0.32
Lifestyle habits questionnaire score		0.58		0.27		0.20		0.68
Abstinence period (days)	0.37 [0.07–0.61]	0.02		0.90		0.76		0.19
Semen ORP (mV/10^6^ sperm/mL)	−0.47 [−0.68–−0.19]	<0.01	−0.32 [−0.57–−0.15]	0.04		0.07		0.57

*β* shows regression coefficient with 95% confidence interval.

**Table 5:** Multiple stepwise regression analyses of clinical and lifestyle parameters related to sperm parameters.

	Total Number (*R*^2^ = 0.42, *p* < 0.01)		Total Motility (*R*^2^ = 0.20, *p* = 0.01)		Progressive Motility (*R*^2^ = 0.13, *p* = 0.01)		DNA Fragmentation (*R*^2^ = 0.14, *p* = 0.01)	
Variables	*β*	*p*	*β*	*p*	*β*	*p*	*β*	*p*
BMI (kg/m^2^)	−0.29 [−0.54–−0.04]	0.02						
Serum d-ROMs (U.CARR)	−0.42 [−0.66–−0.17]	<0.01						
Serum Zn (µg/dL)			0.37 [0.09–0.66]	0.01	0.39 [0.09–0.62]	0.01		
Semen ORP (mV/10^6^ sperm/mL)	−0.34 [−0.59–−0.09]	<0.01	−0.31 [−0.87–−0.17]	0.03				
Serum HDL-C (mg/dL)							0.40 [0.10–0.63]	0.01

The following parameters were utilized as variables for the multiple stepwise regression analyses: Age, BMI, serum d-ROMs, skin AGEs, serum glucose, serum TC, serum HDL-C, serum TG, serum Zn, serum free testosterone, serum AGEs, current smoking habit, current drinking habit, total score in lifestyle habits questionnaire, abstinence period, and semen ORP. *R*^2^ shows the adjusted coefficient of determination. *β* shows the regression coefficient with a 95% confidence interval.

**Table 6:** Metabolic and lifestyle parameters in two subgroups categorized based on baseline serum d-ROM levels.

	Higher Serum d-ROMs Group	Lower Serum d-ROMs Group	*p*
Number	21	20	
Age (years old)	38.0 ± 5.8	37.0 ± 7.0	0.62
Body weight (kg)	74.4 ± 12.1	72.2 ± 11.1	0.55
BMI (kg/m^2^)	24.8 ± 3.2	23.9 ± 3.4	0.43
Waist (cm)	89.2 ± 11.1	86.3 ± 8.9	0.36
Serum d-ROMs (U.CARR)	343 ± 40	267 ± 22	<0.01
Skin AGEs (AF)	1.70 (1.50–1.83)	1.75 (1.70–1.85)	0.44
Serum glucose (mg/mL)	116 (100–133)	102 (97–106)	0.04
Serum TC (mg/dL)	187 (174–199)	189 (172–217)	0.91
Serum HDL-C (mg/dL)	49 ± 15	50 ± 12	0.75
Serum TG (mg/dL)	108 (68–170)	111 (64–159)	0.94
Serum Zn (µg/dL)	83 (76–95)	79 (68–89)	0.29
Serum free testosterone (pg/mL)	11.8 ± 3.0	13.5 ± 4.4	0.15
Serum AGEs (µg/dL)	0.12 (0.11–0.16)	0.12 (0.09–0.17)	0.62
Current smoking habit (%)	28.6	15.0	0.45
Current drinking habit (%)	76.2	75.0	1.00
Lifestyle habits questionnaire score	37.7 ± 5.8	35.9 ± 8.8	0.45
Abstinence period (days)	3.0 (2.0–5.3)	6.0 (3.0–8.0)	0.11
Semen volume (mL)	2.9 ± 0.8	3.7 ± 1.3	0.02
Sperm concentration (10^6^/mL)	114 (66–194)	156 (126–223)	0.07
Total sperm number (10^6^/ejaculate)	352 (158–514)	603 (339–819)	0.02
Sperm total motility (%)	57.1 ± 16.1	64.7 ± 15.8	0.13
Progressive motility in sperm (%)	48.8 ± 16.9	56.6 ± 16.0	0.14
Semen ORP (mV/10^6^ sperm/mL)	0.31 (0.16–0.43)	0.20 (0.12–0.35)	0.30
Sperm DNA fragmentation (%)	5.8 (2.8–9.6)	4.0 (2.6–7.2)	0.37

Data are presented as mean ± SD, median with 25 and 75 percentiles, or percentage as appropriate.

## Data Availability

The original contributions presented in this study are included in the article/Supplementary Material. Further inquiries can be directed to the corresponding author.

## Supplementary Materials

The following supporting information can be downloaded at: https://www.mdpi.com/article/10.3390/jcm13237158/s1, Table S1: The results of Shapiro-Wilk test. Table S2: The detailed results of the lifestyle habits questionnaire. Table S3: Multiple regression analyses including lifestyle factors as mandatory variables. Table S4: Metabolic and lifestyle parameters in two subgroups categorized based on sperm phenotype. Table S5: Raw statistical data of serum d-ROMs and semen ORP levels and sperm phenotype. Table S6: Correlation matrices for serum d-ROMs and semen ORP levels and different sperm phenotypes.
