# Supplementary material for: Impact of Oxidative Stress on Sperm Quality in Oligozoospermia and Normozoospermia Males Without Obvious Causes of Infertility
Source: J Clin Med. 2024 Nov 26;13(23):7158. doi: 10.3390/jcm13237158 (PMC11642397; doi:10.3390/jcm13237158)
Supplement: Supplementary file 1 [file jcm-13-07158-s001.zip › jcm-3311702-supplementary.pdf]

Supplemental Table S1. The results of Shapiro-Wilk test

Parameters	*p*
Age (years old)	0.06
Body weight (kg)	0.82
BMI (kg/m^2)	0.68
Waist (cm)	0.28
Serum d-ROMs (U.CARR)	0.20
Skin AGEs (AF)	0.03
Serum glucose (mg/mL)	<0.01
Serum TC (mg/dL)	<0.01
Serum HDL-C (mg/dL)	0.33
Serum TG (mg/dL)	0.04
Serum Zn (μg/dL)	0.03
Serum free testosterone (pg/mL)	0.53
Serum AGEs (μg/dL)	<0.01
Lifestyle habits questionnaire score	0.20
Abstinence period (days)	<0.01
Semen volume (mL)	0.08
Sperm concentration (10^6/mL)	0.01
Total sperm number (10^6/ejaculate)	<0.01
Sperm motility (%)	0.10
Progressive motility of sperm (%)	0.13
Semen ORP (mV/10^6 sperm/mL)	<0.01
Sperm DNA fragmentation (%)	<0.01

Supplemental Table S2. The detailed results of the lifestyle habits questionnaire.

A. How often do you exercise (30-minute walk or equivalent)?				
1. Not at all	2. No exercise but walk in office or house	3. Once a week	4. 2–3 times a week	5. More than 4 times a week
6 (14.6%)	13 (31.7%)	4 (9.8%)	11 (26.8%)	7 (17.0%)
B. Do you smoke?				
1. Daily smoking for ≥10 years	2. Daily smoking for <10 years	3. Quit smoking within 1 year	4. Quit smoking >1 year ago	5. Never have smoked
7 (17.0%)	2 (4.9%)	8 (19.5%)	2 (4.9%)	22 (53.7%)
C. Do you drink alcohol?				
1. More than 4 times a week	2. 2–3 times a week	3. Once a week	4. Sometimes	5. Never
7 (17.0%)	7 (17.0%)	9 (22.0%)	12 (29.3%)	6 (14.6%)
D. How long do you sleep every day?				
1. Less than 4 hours	2. 4–5 hours	3. 5–7 hours	4. 7–8 hours	5. More than 8 hours
0 (0.0%)	6 (14.6%)	14 (34.1%)	18 (43.9%)	3 (7.3%)
E. Do you feel mental stress?				
1. Strongly agree	2. Agree	3. Undecided	4. Disagree	5. Strongly disagree
2 (4.9%)	13 (31.7%)	20 (48.8%)	5 (12.2%)	1 (2.4%)
F. Do you eat a lot of vegetables?				
1. Strongly disagree	2. Disagree	3. Undecided	4. Agree	5. Strongly agree
3 (7.3%)	8 (19.5%)	12 (29.3%)	16 (39.0%)	2 (4.9%)
G. Do you eat breakfast every morning?				

1. Strongly disagree	2. Disagree	3. Undecided	4. Agree	5. Strongly agree
10 (24.4%)	5 (12.2%)	2 (4.9%)	10 (24.4%)	14 (34.1%)
H. Do you eat moderately? (Do you eat until you are 80% full?)				
1. Strongly disagree	2. Disagree	3. Undecided	4. Agree	5. Strongly agree
6 (14.6%)	10 (24.4%)	7 (17.0%)	16 (39.0%)	2 (4.9%)
I. Do you avoid eating oily food?				
1. Strongly disagree	2. Disagree	3. Undecided	4. Agree	5. Strongly agree
8 (19.5%)	14 (34.1%)	12 (29.3%)	6 (14.6%)	1 (2.4%)
J. Do you avoid eating processed foods?				
1. Strongly disagree	2. Disagree	3. Undecided	4. Agree	5. Strongly agree
6 (14.6%)	11 (26.8%)	8 (19.5%)	13 (31.7%)	3 (7.3%)
K. Do you avoid eating sugary food (cakes and candies)?				
1. Strongly disagree	2. Disagree	3. Undecided	4. Agree	5. Strongly agree
9 (22.0%)	14 (34.1%)	6 (14.6%)	11 (26.8%)	1 (2.4%)
L. Do you eat vegetables at the start of meals?				
1. Strongly disagree	2. Disagree	3. Undecided	4. Agree	5. Strongly agree
4 (9.8%)	7 (17.0%)	5 (12.2%)	15 (36.6%)	10 (24.4%)

Values show the number of the participants with the percentage in parentheses.

Supplemental Table S3. Multiple regression analyses including lifestyle factors as mandatory variables

	Total number ($R^2 = 0.48, p < 0.01$)		Total motility ($R^2 = 0.31, p = 0.01$)		Progressive motility ($R^2 = 0.21, p = 0.06$)		DNA fragmentation ($R^2 = 0.19, p = 0.10$)	
Variables	β	p	β	p	β	p	β	p
Current smoking habit (%)		0.56		0.47				
Current drinking habit (%)		0.68		0.40				
Lifestyle habits questionnaire score		0.28		0.09				
BMI (kg/m^2)	−0.28 [−0.54 – −0.02]	0.04						
Serum d-ROMs (U.CARR)	−0.35 [−0.63 – −0.09]	0.01						
Serum Zn (μg/dL)			0.40 [0.11 – 0.69]	<0.01				
Semen ORP (mV/10^6 sperm/mL)	−0.43 [−0.69 – −0.16]	<0.01	−0.35 [−0.65 – −0.05]	0.02				

The following parameters were utilized as variables for the multiple stepwise regression analyses: Age, BMI, serum d-ROMs, skin AGEs, serum glucose, serum TC, serum HDL-C, serum TG, serum Zn, serum free testosterone, serum AGEs, abstinence period, and semen ORP. Current smoking habit, current drinking habit, and total score of lifestyle habits questionnaire were included as mandatory variables for the regression analyses. R^2 shows the adjusted coefficient of determination. β shows the regression coefficient with 95% confidence interval.

Supplemental Table S4. Metabolic and lifestyle parameters in two subgroups categorized based on sperm phenotype

	Normospermia	Oligospermia	*p*
Number	24	17	
Age (years old)	37.4 ± 7.2	37.6 ± 5.1	0.91
Body weight (kg)	71.5 ± 12.2	75.9 ± 10.2	0.24
BMI (kg/m^2)	23.8 ± 3.8	25.2 ± 2.3	0.18
Waist (cm)	87.5 ± 11.4	88.3 ± 8.2	0.79
Serum d-ROMs (U.CARR)	294 ± 46	324 ± 51	0.06
Skin AGEs (AF)	1.70 (1.55–1.95)	1.80 (1.70–1.80)	0.89
Serum glucose (mg/mL)	102 (95–116)	110 (101–123)	0.12
Serum TC (mg/dL)	187 (172–218)	188 (171–195)	0.88
Serum HDL-C (mg/dL)	50 ± 14	49 ± 14	0.86
Serum TG (mg/dL)	115 (64–159)	106 (68–166)	0.89
Serum Zn (μg/dL)	84 (74–99)	79 (65–88)	0.23
Serum free testosterone (pg/mL)	12.3 ± 4.0	13.1 ± 3.6	0.53
Serum AGEs (μg/dL)	0.11 (0.10–0.16)	0.14 (0.10–0.18)	0.31
Current smoking habit (%)	29.2	11.8	0.17
Current drinking habit (%)	75.0	76.5	0.91
Lifestyle habits questionnaire score	36.5 ± 7.2	37.3 ± 7.8	0.73
Abstinence period (days)	4.5 (3.0–8.0)	2.0 (2.0–6.0)	<0.01
Semen volume (mL)	3.6 (2.8–4.1)	2.7 (2.3–3.3)	<0.01
Sperm concentration (10^6/mL)	205 (137–241)	73 (39–118)	<0.01
Total sperm number (10^6/ejaculate)	636 (531–866)	224 (119–314)	<0.01
Sperm motility (%)	68.7 ± 13.2	49.7 ± 13.5	<0.01
Progressive motility of sperm (%)	60.9 ± 13.2	40.8 ± 14.1	<0.01
Semen ORP (mV/10^6 sperm/mL)	0.20 (0.11–0.29)	0.43 (0.26–0.62)	<0.01
Sperm DNA fragmentation (%)	3.5 (2.3–6.5)	6.5 (3.9–11.4)	0.06

Data are presented as the mean ± SD, median with 25 and 75 percentiles, or percentage as appropriate.

Supplemental Table S5. Raw statistical data of serum d-ROMs and semen ORP levels and sperm phenotype

ID	Serum d-ROMs (U.CARR)	Semen ORP ($mV/10^6$ sperm/mL)	Sperm phenotype
1	234	0.20	Normospermia
2	254	0.34	Normospermia
3	299	0.37	Oligospermia
4	310	0.91	Oligospermia
5	287	0.20	Oligospermia
6	282	0.12	Normospermia
7	285	0.11	Normospermia
8	244	0.29	Normospermia
9	392	0.10	Oligospermia
10	266	0.11	Normospermia
11	298	0.22	Normospermia
12	355	0.60	Oligospermia
13	216	0.12	Normospermia
14	247	0.20	Normospermia
15	283	0.28	Oligospermia
16	292	0.36	Normospermia
17	443	0.36	Oligospermia
18	303	0.31	Normospermia
19	329	0.42	Oligospermia
20	282	1.83	Oligospermia
21	310	0.22	Normospermia
22	399	0.22	Normospermia
23	351	0.44	Oligospermia
24	266	0.04	Normospermia
25	315	0.45	Oligospermia
26	338	0.13	Normospermia
27	365	0.17	Normospermia
28	320	0.20	Normospermia
29	308	0.08	Normospermia
30	282	0.40	Normospermia
31	282	0.17	Normospermia
32	350	0.40	Normospermia
33	283	0.58	Oligospermia
34	270	0.11	Normospermia
35	249	0.19	Normospermia

36	307	0.11	Normospermia
37	325	0.11	Oligospermia
38	319	0.42	Oligospermia
39	245	0.66	Oligospermia
40	369	0.28	Normospermia
41	403	1.00	Oligospermia

Supplemental Table S6. Correlation matrices for serum d-ROMs and semen ORP levels and different sperm phenotypes.

	Serum d-ROM	Semen ORP	Sperm phenotype
Serum d-ROM	1		
Semen ORP	0.20 (*p*=0.21)	1	
Sperm phenotype	−0.30 (*p*=0.06)	−0.53 (*p*<0.01)	1

Values show Spearman's rank correlation coefficient.
